# Correction to “*Clostridium difficile* Peritonitis Complicated by Splenic Rupture and Pelvic Abscess Formation in a Patient Previously on Continuous Ambulatory Peritoneal Dialysis”

**DOI:** 10.1155/crgm/9815752

**Published:** 2026-06-17

R. Yassine, A. Smith, N. Jhaveri, and S. Nanthabalan, “*Clostridium difficile* Peritonitis Complicated by Splenic Rupture and Pelvic Abscess Formation in a Patient Previously on Continuous Ambulatory Peritoneal Dialysis,” *Case Reports in Gastrointestinal Medicine*, 2025, 7775218, https://doi.org/10.1155/crgm/7775218.

In the article, there is an error in Figure 1, in which the arrow indicating the region of free intraperitoneal air is incorrect. Please find the correct Figure 1 below:

**FIGURE 1:**
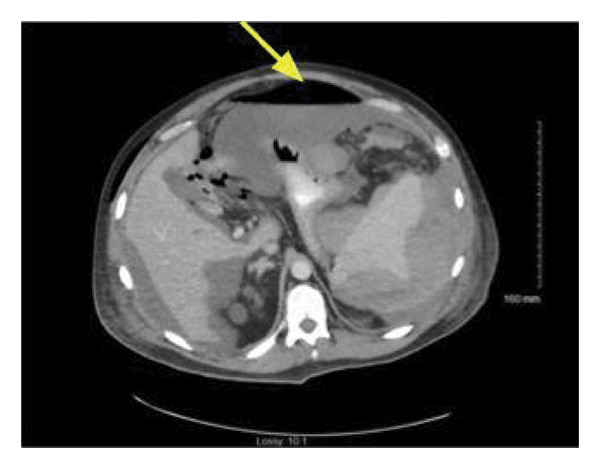
CT abdomen and pelvis with IV contrast shows a moderate volume of intraperitoneal free air mixed with ascites fluid, particularly in the right upper abdomen anteriorly.

We apologize for this error.

